# Becoming a Teenager after Early Surgical Ventricular Septal Defect (VSD) Repair: Longitudinal Biopsychological Data on Mental Health and Maternal Involvement

**DOI:** 10.3390/jcm11237242

**Published:** 2022-12-06

**Authors:** Laura Lang, Jennifer Gerlach, Anne-Christine Plank, Ariawan Purbojo, Robert A. Cesnjevar, Oliver Kratz, Gunther H. Moll, Anna Eichler

**Affiliations:** 1 Department of Child and Adolescent Mental Health, University Hospital Erlangen, Friedrich-Alexander-Universität Erlangen-Nürnberg (FAU), 91054 Erlangen, Germany; 2 Department of Pediatric Cardiac Surgery, University Hospital Erlangen, Friedrich-Alexander-Universität Erlangen-Nürnberg (FAU), 91054 Erlangen, Germany; 3 Department of Pediatric Cardiovascular Surgery, Pediatric Heart Center, University Children’s Hospital Zürich, 8032 Zürich, Switzerland

**Keywords:** congenital heart disease, ventricular septal defect, child development, psychological adjustment, quality of life, stress, adolescence, longitudinal study, mental health, cortisol

## Abstract

Beside somatic strains of congenital heart diseases (CHD), affected children often show developmental impairments in the long term. Ventricular septal defect (VSD) is the most common congenital heart defect and early surgical repair is associated with positive somatic outcomes. However, psychological adjustment is of lifelong relevance. We investigated 24 children with a surgically-corrected isolated VSD and their mothers from primary school (6–9 years) to adolescence (10–14 years) and compared them to controls. Both times, mothers reported child internalizing/externalizing problems, mothers and children rated child quality of life, and children performed neurodevelopmental tests. Adolescents also rated internalizing/externalizing problems themselves, and their hair cortisol levels were analyzed. Maternal anxiety and proactive parenting behavior were considered as moderators. Results revealed no group differences in child neurodevelopment (language, cognition), externalizing problems, and cortisol levels at any time. In reports from mothers, internalizing problems (depression, anxiety) were elevated in children with a VSD at both times—when mothers reported anxiety symptoms themselves. In adolescent reports, VSD patients’ quality of life was increased and internalizing problems were decreased—proactive parenting behavior went along with decreased symptoms in VSD-affected adolescents and with increased symptoms in controls. The findings pronounce the crucial role of parenting behavior and the influence of maternal anxieties on child mental health after surgical VSD repair and might highlight the need for parent-centered interventions.

## 1. Introduction

Congenital heart disease (CHD) is the most frequent birth malformation, with isolated ventricular septal defect (VSD) representing the largest sub-category in children (approximately 37%) [1,2,3,4]. Most isolated VSDs close spontaneously during the first 12 months of life. As a result of modern cardiological and cardiac surgical management, children who require surgery have an excellent long-term outcome regarding their physical abilities [1,5,6,7]. Therefore, the recent research focus has shifted towards examining psychological adjustments in children with CHD. Most of these studies find affected children to be developmentally impaired. especially those with more severe CHD conditions [8,9]. In particular, studies concentrating on neurodevelopmental outcomes showed reduced cognitive development in children with CHD compared to non-affected controls [10,11,12,13] and found that these deficits remain into young adulthood [14,15]. It is also widely understood that children and adolescents with CHD are at an increased risk of internalizing and externalizing problems [12,16,17]. In clinical classification systems such as the DSM-IV, internalizing problems are described as emotional problems that can include disorders such as anxiety and depression, while externalizing or behavior problems comprise disorders such as ADHD or antisocial behavior [18]. In addition, children with CHD are reported to have a higher lifetime prevalence of psychiatric disorders, such as attention deficit/hyperactivity disorder (ADHD) or anxiety disorders [19,20,21]. Still, a recent review found most of these dysfunctions to be high in prevalence but mild [8], which accounts specifically for less severe conditions, as with VSD [22]. Moreover, most of them seem to be more relevant in younger children [23] and might decrease over the individual’s lifetime [24]. The limitations in neurodevelopmental, internalizing, and externalizing function could lead to reduced health-related quality of life (HRQOL) in children with CHD [25,26]. Nevertheless, results on the perceived HRQOL of children with CHD are still inconsistent. Some studies reported satisfactory overall HRQOL with no differences or even better scores compared to non-affected controls [19,27,28,29], whereas other studies found these children to be at a greater risk of impaired HRQOL [30,31,32]. These differences could be attributed to variability in method designs, such as the age of the children at the time of assessment [33] or the severity of CHD [32]. One long-term study exclusively including children with surgically corrected VSDs found increased HRQOL three months after cardiac surgery and even higher scores after at the one-year follow-up [34]. To fully understand psychological development in children with CHD, it seems important to investigate these adjustments on a physiological level, in additional to self-reporting and proxy reports. Some studies found that early stressful life events such as cardiac surgery could lead to alterations to the hypothalamic–pituitary–adrenal (HPA) axis, the key system of stress response, and thus to different patterns of cortisol release [35,36,37]. This dysregulation of the HPA axis could be one mechanism underlying impaired neurodevelopmental and psychopathological outcomes in children [35,36,37]. However, after using a homogeneous sample of children who underwent surgery for a VSD, no differences were found regarding the stress system when compared to non-affected controls.

A CHD does not only affect the child. Besides the impacts of CHD on children’s physical development and later psychological adjustment, the diagnosis, medical treatments, and surgery have a major influence on parents as well [38,39]. Many parents experience tremendous emotional distress (e.g., shock, sadness, guilt); mothers, who are often the primary caregiver, describe immense suffering and anxiety, as well as the burden of caring for a child with CHD [40,41,42]. In addition, mothers of children with CHD have been shown to be less involved in interactions with their child [43], and the parent–child relationship seems to encounter difficulties [44]. Moreover, maternal characteristics such as a lower educational level, anxiety symptoms, or parenting stress were found to be risk factors for increased internalizing and externalizing problems [45,46,47], early delays in cognitive development (e.g., communication difficulties) [10], and lower quality of life (QoL) [28] in children with CHD. Thus, family-related variables (e.g., maternal mental health and parenting style) seem to have an even greater impact on predicting behavioral and cognitive outcomes in children with CHD than disease or surgical factors themselves [48]. In reverse, positive parenting behavior could be a potential protective factor and improve emotional and behavior problems in children with CHD [49]. To summarize, the characteristics of the mother (e.g., parenting behavior, maternal psychopathology) seem to be highly relevant moderators of child development and psychological adjustment and should be considered in studies targeting the longitudinal development of children with CHD. 

## 2. Aims of This Study

Even though there is growing interest in the psychological development of children with CHD [9,12,22], there is still a lack of relevant literature focusing exclusively on children with surgically corrected isolated VSDs. Previously, we compared psychological long-term outcomes between primary school-aged children (t1) who underwent surgery for a VSD in infancy and typically developing children, including assessment of maternal anxiety and proactive parenting behavior as potential moderators [50,51]. The aim of this follow-up study was to reinvestigate these children in adolescence (t2) to reveal potential changes in their psychological adjustments. Specifically, this follow-up study explored the long-term consequences of early surgical VSD correction on children’s neurodevelopment (cognitive development and language), internalizing and externalizing problems (depression, anxiety, attention deficit/hyperactivity disorder (ADHD), and antisocial behavior), HRQOL, and cortisol levels and additionally sought to reveal the role of maternal characteristics (proactive parenting behavior and maternal anxiety) in child development from primary school to adolescence. The development of children who underwent surgery for a VSD was compared to non-affected matched controls. 

*Neurodevelopment*. For primary school-aged children, our study team found no differences in cognitive development (IQ scores) between the groups but did observe weaker language skills in children with VSDs [50]. Since intelligence is a psychological construct that is considered to be stable over time [52], it was hypothesized that adolescents with VSDs do not significantly differ in their cognitive development from typically developing adolescents. In line with the findings of Eichler et al. [50], this study assumed significantly poorer language outcomes in adolescents with VSDs than the comparison group at adolescence. 

*Emotional and behavioral problems.* Regarding the development of psychopathological symptoms, the current literature is still inconsistent regarding whether these symptoms increase or decline over time in children with CHD. At primary school age, Eichler et al. [50] found no differences in internalizing and externalizing behavior problems between the VSD group and the non-affected control group. As adolescence is a critical stage in life when many mental health symptoms appear for the first time [53,54,55], some studies reported adolescents with CHD to have more internalizing and externalizing symptoms and to be at higher risk of a lifetime prevalence of psychiatric disorders compared to non-affected controls [19,21,56]. Moreover, Karsdorp, et al. [57] found that only adolescents with CHD displayed an increased risk of adverse psychological adjustment, which could not be demonstrated in younger children. In contrast, other studies showed that younger children had more internalizing and externalizing problems [23] and that these symptoms might decrease over time [24]. The studies reported above used inhomogeneous samples of children with CHD, including a variety of different disease severities, which could account for the differences in the results. Therefore, this study aimed to investigate whether internalizing and externalizing problems occur in adolescence by using a homogeneous sample of children with an early surgically corrected isolated VSD compared to non-affected controls [51]. 

*HRQOL.* Many studies have targeted the HRQOL of children with CHD, but findings are still heterogeneous. Some authors reported higher HRQOL in adolescence for those with CHD [19,28], while others found their HRQOL to be impaired compared to typically developing adolescents [30,31]. In our study of primary school-aged children [50], we found a trend in mothers of children with a VSD: mothers reported higher HRQOL in their children than the control group (child self-rating did not differ between groups). Since ratings on HRQOL seem to depend on the severity of CHD [32], this study hypothesized, in line with our previous findings [50], that mothers report higher child HRQOL scores compared to non-affected controls in adolescence. 

*Physiological stress regulation.* In addition to self-reporting and proxy reports of child psychological adjustment, we were also interested in physiological stress regulation in children with surgically corrected VSDs compared to typically developing children. Therefore, children’s cortisol levels were assessed. Studies on the association of children with (surgically corrected) CHD and cortisol levels are rare and inconsistent. One study found differences from normal reference values regarding the diurnal variability of salivary cortisol levels in children with CHD [58], while Stonawski et al. [51]—based on our own data—found no differences in diurnal cortisol release between the VSD and control group at primary school age. In adolescence, instead of salivary cortisol, which rather measures acute cortisol production and is sensitive to diurnal rhythm [59], hair cortisol values were used to enable the investigation of cumulative distress over a one-month time period [60]. Since existing studies were heterogeneous in terms of methods and designs and revealed mixed results, this study aimed to investigate whether children with early surgically corrected isolated VSDs differed in their hair cortisol levels from non-affected children. 

*Maternal characteristics as moderators of development.* As mentioned above, maternal characteristics such as parenting behavior and maternal anxiety symptoms can be considered as important moderators of child development, especially in children with surgically corrected VSDs. In the previous study of primary school-aged children, Eichler et al. [50] found mothers’ proactive parenting behavior to be a protective factor that levelled impairments in language development in the VSD group, whereas high maternal anxiety was identified as a risk factor for the development of anxiety symptoms in children with surgically corrected VSDs. Therefore, the final goal of this study was to explore the potential moderating role of maternal parenting behavior and anxiety in children’s psychological adjustment in adolescence.

## 3. Methods

### 3.1. Study Design and Participants

**Figure 1:**
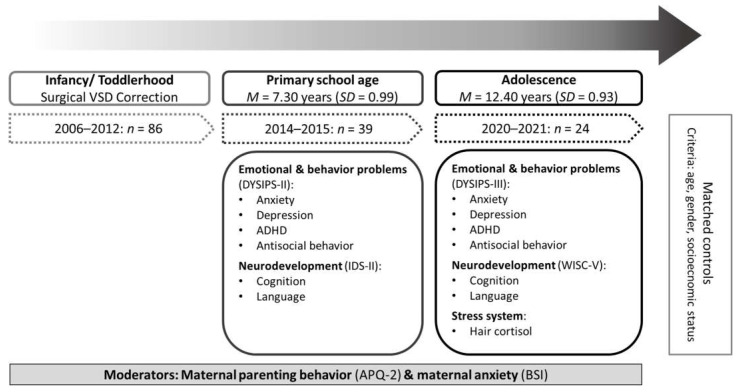
Visual concept model of the study design and variables of interest. *M* = Mean, *SD* = Standard Deviation, *n* = sample size; VSD = ventricular septal defect; DYSIPS = Diagnostic System for Psychiatric Disorders according to ICD-10/DSM-IV [18]; IDS = Intelligence and Development Scales [62]; WISC = Wechsler Intelligence Scale for Children [63]; APQ = Alabama Parenting Questionnaire [64,65]; BSI = Brief Symptom Inventory [66].

Between March 2006 and March 2012, 86 children with an isolated VSD underwent surgery in the Department of Cardiac Surgery at the Erlangen University Hospital, Germany. A total of 26 children were excluded because of genetic syndromes, additional congenital malformations, complex heart defects, or non-cardiological death. Finally, 60 children fulfilled the inclusion criteria. Six families were not available, and fifteen families chose not to participate. Finally, *n* = 39 took part and were invited to a first investigation in 2015 at the Department of Child and Adolescent Mental Health at the Erlangen University Hospital, Germany, to assess their psychological adjustment when they were primary school-aged (t1) (see Eichler et al. [50] for further information). When children were in adolescence, families were reinvited between July 2019 and June 2021 to participate in a follow-up assessment (t2). In total, 15 of the original 39 children (38.5% drop out) did not attend the follow-up assessment, either because they were not interested (*n* = 7) or they were not available (*n* = 8). Children who attended the study at t2 did not differ in sex (*χ*^2^ *=* 0.60, *p* = 0.440), age (*t* = −0.90, *df* = 37, *p* = 0.372), or socioeconomic status (*t* = 0.25, *df* = 37, *p* = 0.801) from the 15 non-attending children. A total of 24 children and their mothers participated in the study at both time points, of which 16 came directly to the Department of Child and Adolescent Mental Health, 4 were visited at home, and 4 participated via mail (non-contact attendance due to the coronavirus pandemic). The VSD group was matched with a non-affected control group (*n* = 24) for sex, age, and socioeconomic status (VSD: *M* = 11.08, *SD* = 2.55; Controls: *M* = 11.63, *SD* = 2.50; for details see Section 3.2.1). The control group was recruited from the Franconian Cognition and Emotion Studies sample [61]. An overview of the study’s design, data assessments, and measurements relevant for this publication can be seen in Figure 1.

Most mothers and fathers had a national origin (87.5–91.7%), with no differences between the VSD group and the controls. Parental education levels were comparable in both groups (VSD group—none/low: mothers 33.4%, fathers 25%; middle: mothers 29.2%, fathers 33.3%; high: mothers 33.3%, fathers 29.2%; 4.1% to 12.5% did not provide any information. Control group— none/low: mothers 25%, fathers 37.5%; middle: mothers 29.2%, fathers 12.5%; high: mothers 45.8%, fathers 50%). Family monthly net income did not differ between the VSD group and the control group (VSD: *M* = 4.05, *SD* = 1.15; Controls: *M* = 4.17, *SD* = 1.17). 

Sample characteristics are shown in Table 1. Age in the VSD group ranged from 10 years 9 months to 14 years 7 months (*M* = 12 years 4 months, *SD* = 0.93), and age in the comparison group ranged between 12 years 9 months and 13 years 8 months (*M* = 13 years 2 moths, *SD* = 0.24). Affected and unaffected children did not differ significantly in sex (VSD: 13 females, 11 males; Controls: 14 females, 10 males; *χ*^2^ = 0.09, *p* = 0.771) or socioeconomic status (*t* = −0.74, *df* = 46, *p* = 0.461) but differed significantly in age (*t* = 4.04, *df* = 46, *p* = 0.001). Children with VSDs and non-affected controls were tested for differences in corporal diseases (auditory, ocular, cutaneous, respiratory, gastrointestinal, cardiovascular, thyroid, endocrine, kidney), mental health (ADHD, anxiety), and medications (antibiotics, corticosteroids, methylphenidate, ketoconazole). No significant differences between the two groups could be demonstrated (*p* = 0.074–1.000). Descriptive sample characteristics are summarized in Table 1.

At both times of measurement, mothers answered standardized questionnaires about their child’s psychopathology and HRQOL, as well as questions about parenting style and maternal anxiety. Children performed a neurodevelopmental test battery and answered a self-report questionnaire on HRQOL. At t2 (adolescence), children were asked about their own psychopathology using standardized questionnaires and child hair samples were taken. The study protocol was approved by the local ethics committee of the University of Erlangen–Nürnberg and conducted in accordance with the Declaration of Helsinki. Mothers gave written informed consent and assent of the children was obtained. 

### 3.2. Measurements

#### 3.2.1. Socioeconomic Status

For assessing families’ socioeconomic status, a sum index was created based on maternal and paternal education level (four categories: <9 [1], 9 [2], 10–12 [3], or 13 [4] years of education), maternal and paternal origin (two categories: international [0] or national [1]), and monthly family income (six categories: less than EUR 1000 [1], EUR 1000–2000 [2], EUR 2000–3000 [3], EUR 3000–4000 [4], EUR 4000–5000 [5], more than EUR 5000 [6]) with a theoretical range from 6 to 16 [50]. 

#### 3.2.2. Neurodevelopment

At t1, the Intelligence and Development Scales (IDS) were applied for measuring children’s neurodevelopment [67] (for more details, see Eichler et al. [50]). At t2, adolescents’ neurodevelopment was assessed using the Wechsler Intelligence Scale for Children—Fifth Edition (WISC-V) [63]. Both procedures represent standardized developmental test batteries for measuring cognitive development and yield IQ (*M* = 100, *SD* = 15) and language competencies (IDS: language score [*M* = 10, *SD* = 3], WISC-V: ‘verbal comprehension’ subtest [*M* = 10, *SD* = 3]). Z-standardization was used in order to transfer the language scales of the IDS and WISC-V into a common unit for further analyses. 

#### 3.2.3. Child Internalizing and Externalizing Outcomes

To measure children’s internalizing and externalizing problems, the Diagnostic System for Psychiatric Disorders (DYSIPS-II) according to ICD-10/DSM-IV for children and adolescents was conducted [18]. With this inventory, different symptoms of psychiatric disorders in childhood and adolescence can be assessed in separate questionnaires. On a four-point Likert scale, with values of “not at all” (0), “a little bit” (1), “to a great extent” (2) and “particularly” (3), both children via self-rating at t2 and mothers via external rating at t1 and t2 assessed statements about the child’s internalizing and externalizing problems during the last six months. In this study, the symptoms of four psychiatric disorders were investigated: anxiety (44 items, e.g., “shows single intense anxiety states that develop within a few minutes”/“I am suddenly overcome by very strong fear, that develops within a few minutes“), depression (29 items, e.g., “seems sad most of the time; often appears close to tears”/“I am sad most of the time and often close to tears“), ADHD (20 items, e.g., “describes a frequently occurring strong feeling of inner restlessness“/“I often run around or climb permanently when it is inappropriate“), and antisocial behavior (38 items, e.g., “I am often angry or offended/“Is often angry or offended“). In all four questionnaires, the mean raw sum was calculated for a total score with a theoretical range of 0.00 (“not at all”) to 3.00 (“particularly”). 

#### 3.2.4. Health-Related Quality of Life

At t1, the mother and child versions of the Revised Quality of Life Questionnaire were used to assess the child’s HRQOL [68] (for more details, see Eichler et al. [50]). Both mothers and children rated the child’s HRQOL at t2 using the German version of the Kidscreen-10 questionnaire, which provides a global score [69]. It comprises ten statements about physical, psychological, and social aspects of HRQOL in relation to the previous week (e.g., “Have you been full of energy?/Was your child full of energy?”), which are scored on a 5-point scale: “not at all/never” (1), “a little bit/rarely” (2), “moderate/sometimes” (3), “quite a bit/often” (4), and “very much/always” (5). The mean raw sum scores of both inventories were transformed into percentage scales ranging from 0 to 100%.

#### 3.2.5. Maternal Anxiety

At both measurement time points, the German version of the Brief Symptom Inventory was used to assess maternal anxiety [66]. The inventory contains 53 items that measure psychological distress and from which nine subscales and a global score (Global Severity Index [GSI]) can be formed. In this study, the 6-item “anxiety” subscale was used as a potential moderator to assess maternal anxiety (*t*-scores, *M* = 50, *SD* 10). The statements of the scale refer to physical and psychological symptoms over the last seven days (e.g., “fearfulness”) and were rated by the mothers on a 5-point Likert scale: “not at all” (0), “a little bit” (1), “quite a bit” (2), “highly” (3), and “very much” (4). 

#### 3.2.6. Maternal Proactive Parenting Behavior

To assess parenting behavior at t1 and t2, the German version of the Alabama Parenting Questionnaire was used as a self-rating inventory for mothers [65,70]. The questionnaire consists of 72 items that form seven subscales. In line with the findings of Eichler et al. [50], this study focused on the subscale “proactive parenting”, which comprises 6 items (e.g., “You explain to your child how to behave well in a certain situation”) and which was rated on a 5-point Likert scale. The mean raw sum score was calculated, with a theoretical range of 1.00 (“almost never”) to 5.00 (“almost ever”).

#### 3.2.7. Hair Cortisol

At t2, to measure children’s hair cortisol concentration (HCC), an at least 1 cm wide strand of hair was cut off near the hairline of the posterior vertex (as close as possible to the scalp) and stored in paper envelopes at 4 °C until analysis. Mothers answered questions about the chemical treatment of their children’s hair, medication intake, infection symptoms, and endocrine diseases over the last six months. In the control group, three children were excluded because of corticosteroid medication and one child was excluded because of chemical hair treatment, resulting in a final sample of 20 controls. From the 24 children with VSDs, 1 child was excluded because their hair sample was missing. There were no relevant medication intake or hair treatment abnormalities in this group. One child had an endocrinological disease but was excluded anyway because no hair cortisol could be extracted (information is listed below). Child body mass index (BMI) was measured as a confounder and one child had an obesity score >30 kg/m^2^ but was still included in the study. 

For HCC quantification, the first proximal centimeter of each sample was cut (1 cm of hair corresponds to about one month of growth) and processed according to the protocol published by Frisch, et al. [71]. Briefly, each sample was subjected to a repeated washing procedure with 2.5 mL isopropanol [72], air-dried, and minced with grinding balls in a ball mill. To calculate the weight of the hair sample, the tar weight (vial and grinding balls) was subtracted from the gross weight (vial, grinding balls, and hair sample). For cortisol extraction, a 4-step method [72,73] with alternating methanol and acetone extraction steps was applied as described by Frisch et al. [71]. The accumulated methanol–acetone supernatants were evaporated at 50 °C and the resulting pellets were stored at −20 °C until analysis. 

To quantify cortisol concentrations, pellets were dissolved in 250 µL of phosphate-buffered saline and cortisol concentrations were determined using a salivary ELISA assay kit (RE52611; IBL International, Hamburg, Germany; intra-assay coefficient of variation (CV): 6.7%) according to the manufacturer’s instructions. Each sample was assayed in duplicate; the mean value and CV of each duplicate were calculated and used for statistical analysis. The cortisol-to-weight ratio was calculated (pg/mg) as follows:

$$\mathrm{Cortisol-to-weight}\;\mathrm{ratio}\;(\mathrm{pg}/\mathrm{mg})=\frac{\mathrm{HCC}\;\left[\frac{\mathrm{ng}}{\mathrm{mL}}\right]\times 0.25\;\mathrm{mL}}{\mathrm{hair}\;\mathrm{sample}\;\mathrm{weight}\;\left[\mathrm{mg}\right]}\times 1000.$$

In the VSD group, four samples had to be excluded because the CV for the duplicate measurement exceeded 30%. Two samples had CV values between 20% and 30% and were not excluded in view of the small sample size, since their inclusion did not lead to significantly different results. Another five samples were excluded as no cortisol could be extracted (*n* = 4) or because the cortisol-to-weight ratio (pg/mg) was too high (outlier value exceeded mean + 3 SD; *n* = 1), resulting in *n* = 14 valid samples for the VSD group. Since the Shapiro–Wilk test revealed that neither HCC mean values nor cortisol-to-weight ratios were normally distributed, both were ln-transformed. Testing sex, age, menarche, and BMI as potential covariates via *t*-tests/Pearson correlations did not reveal any significant association with cortisol concentrations, which is why no covariates were taken into account. Descriptive statistics of children’s hair cortisol are presented in Table 2. For further information see Frisch et al. [71] and Grimm, et al. [74].

## 4. Statistical Analyses

Statistical analyses were performed using the statistical software IBM SPSS Statistics version 28.0 (IBM Corporation, Armonk, NY, USA). All tests were chosen according to individual requirements. In case of non-fulfilment of the specific requirements of single variables, additional non-parametric analyses were conducted. Results of non-parametric analyses showed no differences in statistical significance; therefore, only results of parametric tests are reported in the manuscript. At the beginning of data analyses, potential confounders (age, sex, menarche, BMI, and socioeconomic status) were initially detected by significant Pearson correlations (*r*) (age, BMI, socioeconomic status) or independent *t*-tests (sex, menarche) with regard to child outcomes. Group differences in mother-rated psychological adjustment (depression, anxiety, ADHD, and antisocial behavior, as well as HRQOL) and child neurodevelopment between the VSD group and control group over both periods of measurement were analyzed using repeated measures analysis of covariance (rm ANCOVAs), which were conducted separately for each outcome. Potential moderators were separately entered as covariates (Model A: maternal anxiety moderator; Model B: maternal parenting moderator) into separate t1 (Model A_t1_/B_t1_) and t2 (Model A_t2_/B_t2_) moderator models. As a main time effect was not of interest in the present study, only group effects and group x time interactions are presented in the results. For conducting group comparisons of children’s self-reported problems and HCC, which were only available at t2, ANCOVAs with maternal characteristics as covariates were conducted. All analyses were two-tailed and the level of significance was defined as *p* ≤ 0.05. As our sample size was small, results with a level of significance of *p* ≤ 0.10 were interpreted as trends and partial eta-squared (*η_p_*^2^) values were reported as the measure for effect sizes in (rm) ANCOVAs, with 0.01 ≤ *η_p_*^2^ ≥ 0.05 representing small, 0.06 ≤ *η_p_*^2^ ≥ 0.13 medium, and *η_p_*^2^ > 0.13 large effects [75].

## 5. Results

### 5.1. Preliminary Analyses

Potential effects of sex, age, and socioeconomic status on the dependent variables (cognitive development, language, internalizing and externalizing problems, HRQOL, and hair cortisol) and moderators were tested. Additionally, BMI and menarche were tested as potential covariates with regard to hair cortisol analyses. No significant effects for age, sex, socioeconomic status, BMI, and menarche were observed on the outcome variables and moderators (*p* = 0.052–0.979). However, there was a significant correlation between age and maternal anxiety at t2 (*r* = 0.291, *p* = 0.045) and between socioeconomic status and language at t1 and t2 (t1: *r* = 0.331, *p* = 0.024; t2: *r* = 0.385, *p* = 0.010), cognitive development at t1 (*r* = 0.538, *p* < 0.001), and ADHD symptoms at t1 (*r* = −0.322, *p* = 0.026). The VSD group and non-affected controls did not differ significantly by socioeconomic status. However, as socioeconomic status is frequently related to child neurodevelopment, it was still considered as a confounder when analyzing cognitive development and language scores. 

Maternal anxiety (Table 3) and proactive parenting behavior (Table 4) were considered as potential moderators. In the VSD group, the self-reported maternal anxiety of mothers at t1 did not differ from mothers’ reports of typically developing children (VSD_t1_: *M* = 44.48, *SD* = 8.68; Controls_t1_: *M* = 46.38, *SD* = 7.87; *t*(45) = 0.79, *p* = 0.436). However, mothers of children with VSD showed significantly lower maternal anxiety scores at t2 than mothers of non-affected controls (VSD_t2_: *M* = 39.88, *SD* = 4.52; Controls_t2_: *M* = 46.75, *SD* = 9.49; *t*(46) = 3.20, *p* = 0.002). The two groups showed no differences in maternal proactive parenting behavior at both time points (VSD_t1_: *M* = 3.41, *SD* = 0.62; Controls_t1_: *M* = 3.59, *SD* = 0.49; *t*(44) = 1.05, *p* = 0.298; VSD_t2_: *M* = 3.47, *SD* = 0.67; Controls_t2_: *M* = 3.23, *SD* = 0.51; *t*(42) = −1.34, *p* = 0.187).

### 5.2. Longitudinal Outcomes

As t2 moderator models did not prove to be significant (*p* = 0.108–0.988), only the results with maternal anxiety (Model A_t1_, Table 3) and proactive parenting behavior (Model B_t1_, Table 4) at t1 are presented below. 

#### 5.2.1. Children’s Neurodevelopment

*Cognitive development.* There were no significant main group effects or interaction effects. Affected children’s intelligence did not significantly differ from controls at any time point and was not moderated by any maternal characteristic.

*Language development.* A marginal significant main effect occurred, in which children with VSDs demonstrated lower language scores compared to non-affected controls (Model B _t1_: *F*(1,36) = 3.22, *p* = 0.081, *η_p_*^2^ = 0.08). There was a marginally significant interaction effect (group x time: Model A _t1_: *F*(1,37) = 2.93, *p* = 0.095, *η_p_*^2^ = 0.07; Model B _t1_: *F*(1,36) = 3.23, *p* = 0.081, *η_p_*^2^ = 0.08) at t1, with the VSD group exhibiting weaker language skills than the comparison group (Model A_t1_/B_t1_: *p* = 0.002/0.003). In addition, there was a marginally significant three-way interaction (Model A_t1_: group x maternal anxiety t1 × time: *F*(1,37) = 4.04, *p* = 0.052, *η_p_*^2^ = 0.10). However, post-hoc analyses revealed no significant associations.

#### 5.2.2. Internalizing and Externalizing Behavior Outcomes—Maternal Reports

*Depressive Symptoms.* Regarding internalizing problems, there was a marginally significant main group effect, demonstrating that children with VSDs had higher depression symptoms compared to non-affected controls (Model A_t1_: *F*(1,40) = 4.02, *p* = 0.052, *η_p_*^2^ = 0.09). Additionally, a significant interaction effect between group and maternal anxiety at t1 (Model A_t1_: *F*(1,40) = 5.40, *p* = 0.025, *η_p_*^2^ = 0.12) showed that higher maternal anxiety at t1 was associated with higher depression symptoms in the VSD group, both at t1 and t2 (t1: *r* = 0.607, *p* = 0.002, t2: *r* = 0.645, *p* = 0.002). This interaction remained equally strong over both points of time and was not found in the control group. 

*Anxiety*. There was a significant main group effect: children with VSDs had higher anxiety symptoms than non-affected controls (Model A_t1_: *F*(1,39) = 4.71, *p* = 0.036, *η_p_*^2^ = 0.11). Furthermore, a significant interaction effect between group and maternal anxiety at t1 (Model A_t1_: *F*(1,39) = 6.84, *p* = 0.013, *η_p_*^2^ = 0.15) was found. Post-hoc analyses showed higher maternal anxiety at t1 was associated with higher anxiety symptoms in children in the VSD group, both at t1 and t2 (t1: *r* = 0.673, *p* < 0.001; t2: *r* = 0.475, *p* = 0.034). Conversely, the relationship between children’s anxiety scores and maternal anxiety symptoms at t1 was not significant in the control group. 

*ADHD.* Regarding ADHD symptoms, there was no significant main group effect. A marginally significant interaction effect between group and time (Model B_t1_: *F*(1,42) = 3.01, *p* = 0.090, *η_p_*^2^ = 0.07) demonstrated that ADHD symptoms decreased over time and that the difference in ADHD symptoms between t1 and t2 was larger in the VSD group (*p* < 0.001) than in the control group (*p* = 0.003). Additionally, there was a marginally significant three-way interaction (Model B _t1_: group x proactive parenting behavior t1 × time: *F*(1,42) = 2.89, *p* = 0.096, *η_p_*^2^ = 0.06). However, post-hoc analyses revealed no significant associations. 

*Antisocial behavior.* There were no significant main group or interaction effects. 

#### 5.2.3. Health-Related Quality of Life (HRQOL)

*Maternal ratings.* A marginally significant main effect for groups could be demonstrated: children with VSDs showed higher HRQOL scores than typically developing children (Model B_t1_: *F*(1,39) = 2.94, *p* = 0.095, *η_p_*^2^ = 0.07). A slightly significant interaction effect (Model B_t1_: group × proactive parenting behavior t1: *F*(1,39) = 3.82, *p* = 0.058, *η_p_*^2^ = 0.09) and a slightly significant three-way interaction effect (Model B _t1_: group x proactive parenting behavior t1 × time: *F*(1,39) = 3.90, *p* = 0.055, *η_p_*^2^ = 0.09) were found. Post-hoc analyses revealed that children with VSDs showed significantly higher HRQOL scores at t1 (*p* = 0.015) but not at t2. Further, it was demonstrated that, in the control group, more pronounced proactive parenting behavior at t1 was significantly associated with lower scores in HRQOL at t1 (*r* = −0.443, *p* = 0.034). This association was no longer significant at t2. No significant correlations between proactive parenting behavior and HRQOL occurred in the VSD group. 

*Child ratings.* A significant main group effect could be demonstrated: children with VSDs reported higher HRQOL compared to non-affected controls (Model B _t1_: *F*(1,35) = 4.52, *p* = 0.041, *η_p_*^2^ = 0.11). Additionally, there was a significant interaction effect (Model B _t1_: group x proactive parenting behavior t1: *F*(1,35) = 4.60, *p* = 0.039, *η_p_*^2^ = 0.12), with post-hoc analyses demonstrating that, in the control group, more pronounced proactive parenting behavior at t1 was related to significantly lower child self-reports in HRQOL at t1 (*r* = −0.455, *p* = 0.029) and marginally lower HRQOL scores at t2 (*r* = −0.361, *p* = 0.091). In the VSD group, no significant associations between proactive parenting behavior at t1 and self-reported HRQOL could be found at both times of measurement.

### 5.3. Cross-Sectional Outcomes

#### 5.3.1. Internalizing and Externalizing Behavior Outcomes—Children’s Reports

*Depressive symptoms.* A significant main effect occurred, showing that children with VSDs reported less depressive symptoms than their non-affected peers (Model B_t1_: *F*(1,38) = 6.56, *p* = 0.015, *η_p_*^2^ = 0.15). Furthermore a significant interaction effect between group and maternal proactive parenting behavior at t1 was found (Model B_t1_: *F*(1,38) = 6.87, *p* = 0.013, *η_p_*^2^ = 0.15). In the control group, more pronounced maternal proactive parenting behavior was significantly correlated with higher reported depressive symptoms (*r* = 0.424, *p* = 0.044). No significant association between maternal proactive parenting behavior and depressive symptoms was found in the VSD group.

*Anxiety*. A slightly significant main group effect (Model B_t1_: *F*(1,37) = 3.98, *p* = 0.053, *η_p_*^2^ = 0.10) was detected: children with VSDs reported marginally lower anxiety symptoms than typically developing children. Additionally, a significant interaction effect (Model B_t1_: group × proactive parenting behavior t1: *F*(1,37) = 4.42, *p* = 0.042, *η_p_*^2^ = 0.11) was found. Post-hoc analyses showed that, in the VSD group, higher maternal proactive parenting behavior at t1 was significantly associated with lower anxiety ratings at t2 (*r* = −0.460, *p* = 0.041). There was no significant correlation between maternal proactive parenting behavior and anxiety symptoms in the control group. 

*ADHD.* Self-ratings on ADHD showed no significant main group or interaction effects. 

*Antisocial behavior.* A slightly significant main group main occurred (Model A_t2_: *F*(1,41) = 3.26. *p* = 0.078, *η_p_*^2^ = 0.07): children with VSDs reported higher antisocial behavior than non-affected controls. Additionally, a marginally significant interaction effect between group and maternal anxiety at t2 could be demonstrated (Model A_t2_: *F*(1,41) = 3.34, *p* = 0.075, *η_p_*^2^ = 0.08). However, post-hoc analyses revealed no significant associations.

#### 5.3.2. Children’s Hair Cortisol 

Results are shown in Table 5. A slightly significant main group effect was observed (Model B_t2_: *F*(1,28) = 3.09, *p* = 0.090, *η_p_*^2^ = 0.10), demonstrating that children with VSDs had lower hair cortisol values compared to non-affected controls. Marginally significant interaction effects could be shown (Model B_t1_: *F*(1,28) = 3.73, *p* = 0.064, *η_p_*^2^ = 0.12; Model B_t2_: *F*(1,28) = 3.94, *p* = 0.057, *η_p_*^2^ = 0.12). In the control group, higher maternal proactive parenting behavior at t1 was significantly correlated with higher hair cortisol levels at t2 (*r* = 0.451, *p* = 0.053). In the VSD group, higher maternal proactive parenting behavior at t2 was significantly associated with children’s lower hair cortisol values at t2 (*r* = −0.804, *p* = 0.002).

## 6. Discussion

The aim of this follow-up study was to explore the long-term consequences of early surgical VSD correction on children’s neurodevelopment, internalizing and externalizing problems, HRQOL, and hair cortisol levels from primary school age to adolescence, as well as to additionally reveal the role of maternal characteristics (proactive parenting behavior and maternal anxiety) in child development. Children with early surgically repaired isolated VSDs were examined in comparison to a matched, non-affected control group.

### 6.1. Long-Term Psychological Adjustment

*Neurodevelopment*. This study hypothesized no differences in cognitive development between children with VSDs and non-affected controls except for poorer language outcomes in the VSD group. In line with the results when children were primary school-aged, as already published by Eichler et al. [50], this study found no differences in cognitive development between children with VSDs and typically developing children in adolescence. Other studies reported an association between the severity of CHD and the degree of cognitive impairment and predicted cognitive outcomes in adolescence [15,76,77]. As VSDs are considered to be a mild form of CHD, our result is in line with previous findings showing that cognitive development in mild CHDs seems to be comparable to the non-affected population, also in adolescence [57]. In this study, children with VSDs showed tendentially lower language scores compared to non-affected controls at primary school age (as already shown by Eichler et al. [50]), though not in adolescence. Fourdain, et al. [78] also found no global neurodevelopmental impairment in children with CHD, but a discrepancy between language and cognitive development was observed. The authors discussed delayed development in the frontal and temporal cortical areas (areas associated with speech production and comprehension) and prenatal and postnatal white matter alterations as causative factors of specific language impairment. This effect is not very stable, as it could only be shown when maternal proactive parenting behavior was considered as a potential moderator. Therefore, the results must be replicated. However, contrary to our initial assumption, the finding of this study suggests that the difference in language abilities between children with VSDs and typically developing children evens out during adolescence. Since this effect occurred when both moderators were taken into account, it could be considered as stable. Additionally, a moderate effect showed that higher maternal anxiety at t1 seemed to only be a risk factor for language impairment in children with VSDs when they were primary school-aged, and not during adolescence. Nevertheless, the result should be replicated as it could only be shown in one model (when considering maternal anxiety as a potential moderator).

*Emotional and behavioral problems.* The present study aimed to investigate whether VSD-affected children show more internalizing and externalizing symptoms than non-affected controls in adolescence while not showing differences during childhood (see Eichler et al. [50]). Mothers of children with VSDs reported at least marginally more internalizing problems (depression and anxiety) in their children during childhood and adolescence compared to mothers of non-affected controls, which is in line with most studies on this matter [23,56]. In this context, in the VSD but not in the control group, maternal anxiety at primary school age t1 acted as a significant moderator of child development. When mothers had higher anxiety scores, they also described their children to have more depressive and anxiety symptoms, both at primary school age and in adolescence. This finding is interesting as only in the VSD group (and not in controls) did maternal anxiety levels decrease from primary school age to adolescence. In addition, regardless of group, all maternal anxiety levels were in a normal range and not clinically apparent. However, even at this low level of maternal anxiety, it seems to be relevant for children’s and adolescents’ mental health after early surgical VSD correction. There might be at least two explanations for these findings. First, elevated maternal anxiety might lead to higher internalizing symptoms in the vulnerable group of children with surgically corrected VSDs. In line with this thought, Eichler and colleagues [50] found higher maternal anxiety to be a risk factor for developing more anxious symptoms in children with surgically corrected VSD when they were primary school-aged, and maternal anxiety thus served as a risk factor. Second, mothers who have had the experience of having a child with a VSD and associated cardiac surgery and medical treatments and who have experienced higher anxiety symptoms might be subject to different perceptions of children’s internalizing symptomatology than mothers of typically developing children. Such an altered perception due to their own anxiety symptomatology could lead to a stronger rating of child internalizing behavior problems. To sum up, our findings support the assumptions of several current studies that emphasize the important role of maternal characteristics (e.g., parenting stress, maternal mental health) in the psychological adjustment of children with VSDs [47,79,80]. With regard to our results, this effect seems to outlast childhood and remain until adolescence. The group difference in anxiety symptoms occurred under both moderators and can therefore be interpreted as stable. The other results on internalizing problems should be replicated, as they could only be demonstrated when maternal anxiety was taken into account. 

Mothers’ reports on their children’s externalizing problems (ADHD and antisocial behavior) revealed no differences between the VSD and control group. This result is contrary to various studies showing an increased risk of ADHD for children with CHD [12,22]. A current review of behavioral problems in children with CHD found these children to be at greater risk of internalizing but not externalizing problems and discussed method differences in this context (e.g., type of questionnaire) as a potential cause [81]. Methodological differences in assessing internalizing and externalizing problems might not be relevant in our study as we used symptom-specific subtests of one questionnaire. Dahlawi et al. [23] found younger children with CHD to be at greater risk of behavior problems compared to older children, which is in line with the results of this study showing that ADHD symptoms decreased over time and that the difference in ADHD symptoms between primary school age and adolescence was larger in the VSD group. An explanation for this matter could be a temporal association between the occurrence of risk factors (e.g., children’s physical health) and externalizing behavior problems, as found by Kjeldsen, et al. [82]. If the surgical correction for the VSD is performed in early childhood, it may no longer account for the externalizing behavior problems in adolescence. Additionally, a highly engaged parenting style at t1 was associated with lower ADHD symptoms in primary school-aged children. This correlation was not significant but showed a moderate effect size, indicating the potential protective role of proactive parenting behavior on ADHD symptoms in children with VSDs. The results regarding externalizing problems should be replicated, as they could only be demonstrated when maternal proactive parenting behavior was considered as a potential moderator. The model is therefore only slightly stable. 

*HRQOL.* In line with our assumptions and the already published results for primary school-aged children [50], mothers of children with VSDs reported higher HRQOL in their children compared to mothers of typically developing children. Several studies have reported on decreased HRQOL as a function of the severity of the CHD; in less severe CHDs, higher HRQOL compared to non-affected control groups might be expected [19,29,32]. Conversely, in our sample of children who underwent surgical VSD correction, even higher HRQOL than in typically developing children was found. Eichler et al. [50] explained that a mother’s experience of having a newborn with a serious disease might influence their reference level in terms of their child’s quality of life. As children with VSDs also showed higher self-ratings for HRQOL than controls, this modified reference level may also account for these self-report differences. 

Maternal proactive parenting behavior (assessed when children were primary school-aged) was found to influence the development of children’s HRQOL. In the group with typically developing children, both mothers and children reported an association between maternal proactive parenting behavior and HRQOL; higher proactive parenting behavior was associated with lower HRQOL. In contrast, this relation did not show up in the VSD group. Maternal proactive parenting behavior is described as greater engagement in mother–child interactions, including more sensitive, stimulating, and supporting behavior [70]. We suppose that children with VSDs, who have experienced a life-threatening disease in their early childhood, require higher maternal proactive parenting behavior to develop similarly to their non-affected peers [50]. Thus, highly proactive parenting behavior might be a protective factor in at-risk contexts, such as in the case of early surgical VSD corrections, and may be prone to an overdose effect in unaffected controls, especially in adolescence. Whether maternal proactive parenting behavior actually has a different function for children with an early surgically corrected VSD and non-affected children should be the subject of future studies. The effects on children’s HRQOL only occurred when maternal proactive parenting behavior was taken into account and should therefore be replicated in future studies. 

In summary, the results of this study showed that the long-term effects of an early surgically corrected VSD on psychological adjustment in children and adolescents must be considered in a differentiated manner. The sole presence of a VSD does not automatically lead to behavior problems and lower HRQOL in children and adolescents. We could observe that internalizing behavior problems seemed to be present and stable over time, especially when maternal anxiety during primary school was high. By contrast, there was no evidence of the presence of externalizing behavior problems in adolescence. However, there was an indication that high maternal engagement in mother–child interaction could act as a protective factor regarding ADHD symptoms in children with VSDs.

### 6.2. Children’s Self-Reported Psychological Adjustment and Stress System in Adolescence

This study aimed to investigate if VSD-affected children differ in terms of their self-reported psychological adjustment and hair cortisol values from typically developing children. In adolescence, children with VSDs reported lower depression and anxiety symptoms during adolescence compared to typically developing children. Several studies found similar findings by demonstrating no difference in self-reported psychological adjustment between adolescents with VSDs and non-affected controls [83,84]. As already mentioned, the intensity of psychopathological symptoms is related to the severity of the CHD, as children with more severe CHD are at higher risk of developing psychosocial impairments [8].

Interestingly, the control group showed a comparable interaction with maternal proactive parenting behavior at t1 in terms of depressive symptoms, as was already shown for HRQOL. Non-affected children reported higher depressive symptoms in adolescence when mothers showed a pronounced engagement in mother–child interaction. In childhood, high maternal proactive parenting behavior is important for child development and leads to stronger mother–child attachment and a more sensitive perception of one’s own emotions, as described above [70,85]. In adolescence, as a natural effect of puberty, children detach themselves from their parents and become more independent [86], which is why conflicts between children and parents increase [87]. Therefore, adolescents who experienced pronounced maternal involvement in childhood have to actively detach themselves from their close maternal bond, which is a developmentally appropriate step towards reaching individualization. Thus, high maternal engagement might be perceived as a stress factor in typically developing adolescents in this study. Further, for adolescents with VSDs, an opposite association was demonstrated. When mothers were more engaged in mother–child interaction, children with VSDs reported lower anxiety symptoms in adolescence. When mothers of children with VSDs managed to control the fears surrounding their children and establish a proactive parenting style, this had a positive effect on their children’s development, (such as with language outcome results in a study by Eichler et al. [50]). In adolescence, the following questions arise: Why do children with VSDs not show the same developmental steps as their non-affected peers? Why do children with VSDs not experience the same difficulties and why is pronounced maternal engagement not perceived as a stress factor in adolescence? Developmental gaps could be an explanation. It is possible that this developmental stage is delayed in children with VSDs or is omitted altogether. Another explanation might lay in the age difference between the VSD and control group, as the non-affected children were older than those in the VSD group. Children with VSDs may also have a closer bond with their mothers because of their condition. In any case, future studies should be dedicated to these questions. 

Regarding self-ratings for externalizing problems, adolescents with VSDs showed slightly higher symptoms of antisocial behavior compared to controls, while there were no differences in ADHD. Since antisocial behavior is mainly associated with the onset of puberty, it is not surprising that the differences between children with VSDs and controls only emerge in adolescence [88]. It is also known that early adverse life events are associated with antisocial behavior in adolescence [89,90], and several studies were able to show more pronounced externalizing problems in adolescents with VSDs compared to non-affected controls [46,91]. In this context, we also identified maternal anxiety shaping self-ratings of antisocial behavior in adolescents with surgically corrected VSDs. High maternal anxiety (assessed when children were in primary school) was related to lower symptoms of antisocial behavior. Conversely, this association was not found in the control group. This might be explained by the authors of [92], who suggested that mothers with more anxiety symptoms use a more overprotective parenting style. In turn, overprotective behavior could serve a positive developmental function in children with a chronic disease [93]. Therefore, future research should focus on how maternal characteristics might shape child development differently in children with and without CHD. 

In this study, both the VSD and the control group had hair cortisol levels in the normal range. Interestingly, children with VSDs showed marginally lower hair cortisol values compared to the control group. Cortisol levels in the normal range for children with VSDs have also been observed in other studies [51,94] and indicate development comparable to non-affected peers. Golub, et al. [95] demonstrated that children with a chronic disease display hypocortisolism. Even though children with a surgically corrected VSDs have to be regarded as somatically healthy and not as chronically ill, our sample might also react with comparable hypocortisolism. In the VSD group, children’s self-reports on their better psychological adjustments fit very well with the findings regarding their lower cortisol levels in comparison to typically developing children. Additionally, in children with VSDs, lower cortisol levels were slightly associated with more pronounced maternal proactive parenting behavior, while the association was reversed in the control group. In line with the results regarding internalizing behavior problems, a highly engaged parenting style seems to be a stress factor for non-affected adolescents, which is a developmentally appropriate step towards reaching individualization. Again, further studies are needed to clarify the following question: Why do adolescents with VSDs not show the same development steps as their peers?

In general, the ratings provided by adolescents with VSDs regarding their internalizing and externalizing problems did not match their mothers’ ratings in this study. The discrepancy in self-reports and proxy ratings found in our study corresponds to the study of Spijkerboer, et al. [96], who showed that parents of children with CHD reported higher internalizing behavior problems than their children, with children rating themselves comparably to (or even better than) their non-affected peers. The discrepancy between self-reports and proxy ratings of behavior problems has been demonstrated in several studies, with the difference explained by increased autonomy and therefore less behavioral observation from parents [97,98]. These findings emphasize the need for both self-reports and proxy ratings when trying to realize a better understanding of psychological adjustment in children with VSDs. 

All effects on children’s psychological adjustments and hair cortisol values occurred under only one of the two moderators, not in both models. Therefore, the effects should be interpreted as slightly stable and should be replicated.

## 7. Strengths and Limitations 

A clear strength of the present study is the multi-level approach towards data collection. In addition to maternal ratings, we also collected self-ratings in order to obtain several perspectives on the children’s psychological adjustments and relied on standardized tests for assessing children’s neurocognitive development, which were performed by trained researchers. Further, children’s hair cortisol levels were evaluated in order to gain information on children’s physiological stress system. This provided comprehensive insight into the children’s psychological development. 

An additional strength of this study is the longitudinal approach to assessing children’s psychological adjustment over time in a homogeneous group of children with an isolated surgically corrected VSD and a matched non-affected control group. Longitudinal studies always carry the risk of participants dropping out and may result in smaller sample sizes. For example, in this case, it could be that mothers who were concerned about the development of their children chose to continue participating in the study more than mothers who were less concerned, which may exaggerate the results. Therefore, results should be interpreted carefully. The consistently medium to high effect sizes indicate the practical relevance of our results and should be replicated in future studies with larger sample sizes. 

One limitation of the present study might lay in the age difference between the VSD and control group, as the non-affected children were older than the VSD group. However, the control group was still well matched regarding other sample characteristics. 

Furthermore, an important issue to discuss is the approach taken to testing our hypotheses. To analyze one hypothesis, we conducted four (rm) ANCOVAs separately for each outcome variable and moderator. We chose this data analyzing approach by including as few variables as possible in each model to increase test power due to the small sample size. In the case of multiple testing, α-levels have to be corrected. However, we decided against an alpha correction because of the explorative character of our study. The results have to be interpreted cautiously while taking effect size measures into account. For this reason, the presented findings should be replicated with a larger sample.

Another limitation is not including data from fathers, which leads to a one-sided parental perspective and hides potential protective or risk factors. Future studies should include fathers’ perceptions of their children’s health and explore the role of paternal characteristics in child development for children with surgically corrected VSDs.

## 8. Conclusions

The results of this study indicate that children with an early surgically repaired VSDs have the potential for age-typical development in terms of intellectual abilities, psychological adjustment, and stress response. This seems to remain stable over time, as the results of this follow-up study replicated findings from primary school-aged children with surgically corrected VSDs (cf. [50]). In general, the sample of this study was clinically not suspicious. All outcomes for VSD-affected and unaffected children were within a normal non-pathological range. There are some relevant factors supporting the age-typical development of children after an early surgical VSD repair, such as lower maternal anxiety symptoms and proactive parenting behavior. The relevance of these factors was already shown during childhood in a previous cohort [50], and the effect lasts into adolescence. Maternal proactive parenting behavior proved to be an important factor associated with positive psychological adjustment in children with VSDs and is thus a promising approach for future interventions. Conversely, mothers of children with surgically repaired VSDs still reported heightened internalizing problems in adolescence. Even though the amount of maternal anxiety experienced by mothers of children with surgical VSD correction decreased from primary school age to adolescence, relatively high levels of maternal anxiety still acted as an amplifying factor in this context. Based on the differences between self-reports and proxy ratings of children’s psychopathological symptoms and the association found between emotional and behavioral problems and maternal anxiety, one can deduce that the psychological long-term consequences of an early corrected VSD are more likely to manifest in the mothers, instead of in the children. Mothers were exposed to enormous trauma through diagnosis of and early operation on their child. Therefore, they might find it difficult to let go and have confidence in the positive psychological and somatic development of their children. This highlights the importance of further studies on the psychological well-being of mothers of children with CHD and how they can be adequately supported from the beginning.

## Figures and Tables

**Table 1:** Descriptive Data.

	VSD (*n* = 24)		Controls (*n* = 24)		Statistics	
	*M/n*	*SD/%*	*M/n*	*SD/%*	*t/χ* ^2^	*d/φ*
**Mother-related**						
Anxiety_t1_	44.48	8.68	46.38	7.87	−0.79	−0.23
Anxiety_t2_	39.88	4.52	46.75	9.49	3.20	0.93 **
Parenting_t1_	3.41	0.62	3.59	0.49	1.05	0.31
Parenting_t2_	3.47	0.67	3.23	0.51	−1.34	0.41
**Child-related**						
Child’s age (y)	12.40	0.93	13.19	0.24	4.04	1.17 **
Sex						
Male	11	45.80	10	41.70	0.09	−0.04
Female	13	54.20	14	58.30		
Physical development						
BMI	20.58	7.29	20.05	3.14	−0.32	−0.09
School level						
low	9	37.50	4	16.70	2.65	0.24
middle	7	29.20	9	37.50		
high	8	33.30	11	45.80		

Note. ** *p* < 0.01. Continuous variables are reported as *M* (*SD*) and group differences were tested using a *t*-test with Cohen’s *d* as the effect size measure; categorical variables are reported with *n* (*%*) and group differences were tested using the *χ*^2^ test with the phi-coefficient *φ* as the effect size measure. Single missing data are demonstrated by reduced degrees of freedom (*df*): Anxiety *df* = 45–46, Parenting *df* = 42–44, Child’s age *df* = 46, Sex *df* = 1, BMI *df* = 45, School level *df* = 2. Anxiety = maternal anxiety: German version of the Brief Symptom Inventory [66]; Parenting = proactive parenting behavior: German version of the Alabama Parenting Questionnaire [64,65]; BMI = body mass index; VSD = ventricular septal defect.

**Table 2:** Hair Analysis Results: Descriptive Data.

	VSD				Controls				Statistics (*df* = 32)		
	*n*	*M (SD)*	*Min*	*Max*	*n*	*M (SD)*	*Min*	*Max*	*t*	*p*	*d*
HCC, ng/mL	14	0.11 (0.08)	0.02	0.31	20	0.19 (0.13)	0.04	0.51	2.04	0.050	0.71
Hair sample weight, mg	14	19.34 (5.71)	9.80	29.60	20	21.97 (10.57)	3.06	56.90	0.85	0.404	0.30
Cortisol-to-weight-ratio, pg/mg	14	1.41 (0.87)	0.33	3.45	20	2.33 (1.51)	0.60	7.20	2.04	0.050	0.71

Variables are reported as *M* (*SD*) and group differences were tested using a *t*-test with Cohen’s *d* as the effect size measure. HCC = hair cortisol concentration; VSD = ventricular septal defect.

**Table 3:** Repeated Measures ANCOVA-Tested Differences between the VSD Group and Controls. Covariate: Maternal Anxiety t1 (Model A_t1_).

	VSD		Controls		Statistics *F* (*η*^2^*_p_)*							
	*M*	*SD*	*M*	*SD*	Group		Group × Time		Group × Anxiety t1		Group × Anxiety t1 × Time	
**Neurodevelopment**												
Cognitive_t1_	97.11	12.19	105.17	11.34	0.08	(0.00)	0.13	(0.00)	0.59	(0.02)	0.12	(0.00)
Cognitive_t2_	99.72	11.42	107.83	10.04								
Language_t1_	−0.42	1.06	0.46	0.78	0.44	(0.01)	2.93 +	(0.07)	0.02	(0.00)	4.04 +	(0.10)
Language_t2_	−0.26	1.12	0.18	0.67								
**Maternal psychopathology rating**												
Depression_t1_	0.17	0.20	0.10	0.16	4.01 ^+^	(0.09)	0.07	(0.00)	5.40 *	(0.12)	0.13	(0.00)
Depression_t2_	0.15	0.21	0.10	0.12								
Anxiety_t1_	0.30	0.32	0.20	0.17	4.71 *	(0.11)	0.28	(0.01)	6.84 *	(0.15)	0.39	(0.01)
Anxiety_t2_	0.18	0.27	0.09	0.14								
ADHD_t1_	0.62	0.47	0.60	0.45	1.04	(0.02)	0.29	(0.01)	1.15	(0.03)	0.19	(0.00)
ADHD_t2_	0.38	0.39	0.42	0.40								
Antisocial_t1_	0.17	0.14	0.20	0.17	0.54	(0.01)	0.00	(0.00)	0.52	(0.01)	0.01	(0.00)
Antisocial_t2_	0.11	0.10	0.11	0.09								
**Quality of life**												
Mother_t1_	86.35	7.11	80.30	9.06	2.06	(0.05)	1.11	(0.03)	1.55	(0.04)	2.12	(0.05)
Mother_t2_	64.60	9.86	64.00	7.89								
Child_t1_	74.15	12.96	74.24	9.29	0.31	(0.01)	0.48	(0.01)	0.27	(0.01)	0.62	(0.02)
Child_t2_	63.25	9.77	62.00	8.94								

Note. ^+^ *p* < 0.10, * *p* < 0.05. VSD = ventricular septal defect. Neurodevelopment: t1, Intelligence and Development Scales, IDS [67]; t2, Wechsler Intelligence Scale for Children—Fifth Edition, WISC-V [63]; Cognitive = cognitive development: *M* = 100, *SD* = 15; Language: z-standardization was used in order to transfer the IDS and WISC-V values into a common unit, *M* = 0, *SD* = 1. Maternal psychopathology ratings at t1 and t2: Diagnostic System for Psychiatric Disorders according to ICD-10/DSM-IV, DYSIPS [18]—0, ‘not at all’; 1, ‘a little bit’; 2, ‘to a great extent’; 3, ‘particularly’. ADHD = attention deficit/hyperactivity disorder, Antisocial = antisocial behavior. Quality of life: t1, Revised Quality of Life Questionnaire [68]; t2, German version of the Kidscreen-10 questionnaire [69] (0 to 100% quality of life). Sample size (*n*): VSD: Cognitive/Language *n* = 18; maternal psychopathology ratings, Depression/Anxiety/Antisocial *n* = 20, ADHD *n* = 23; quality of life, Mother *n* = 20, Child *n* = 16. Controls: Cognitive/Language *n* = 24; maternal psychopathology ratings, Depression/ADHD/Antisocial *n* = 24, Anxiety *n* = 23; quality of life, Mother/Child *n* = 24. Covariates—Cognitive/Language: socioeconomic status. Degrees of freedom in rm ANCOVAs (*df_F_*): Cognitive/Language *df_F_* = 37, Depression *df_F_* = 40, Anxiety *df_F_* = 39, ADHD *df_F_* = 43, Antisocial *df_F_* = 40, Quality of life Mother *df_F_* = 40, Quality of life Child *df_F_* = 36. *dfH* = 1. *η*^2^*_p_*, partial eta-squared, effect size measure: ≥0.01 small effect, ≥0.06 medium effect, ≥0.14 large effect.

**Table 4:** Repeated Measures ANCOVA-Tested Differences between the VSD Group and Controls. Covariate: Maternal Parenting t1 (Model B _t1_).

	VSD		Controls		Statistics *F* (*η*^2^*_p_*)							
	*M*	*SD*	*M*	*SD*	Group		Group × Time		Group × Parenting t1		Group × Parenting t1 × Time	
**Neurodevelopment**												
Cognitive_t1_	97.11	12.19	104.38	11.46	1.60	(0.04)	1.41	(0.04)	0.82	(0.02)	1.37	(0.04)
Cognitive_t2_	99.72	11.42	107.39	10.02								
Language_t1_	−0.42	1.06	0.50	0.78	3.22 ^+^	(0.08)	3.23 ^+^	(0.08)	1.89	(0.05)	2.51	(0.07)
Language_t2_	−0.26	1.12	0.20	0.68								
**Maternal psychopathology rating**												
Depression_t1_	0.17	0.20	0.10	0.16	2.83	(0.07)	0.24	(0.01)	2.30	(0.06)	0.17	(0.00)
Depression_t2_	0.15	0.21	0.10	0.12								
Anxiety_t1_	0.30	0.32	0.20	0.17	3.12 ^+^	(0.08)	0.34	(0.01)	2.42	(0.06)	0.30	(0.01)
Anxiety_t2_	0.18	0.27	0.10	0.14								
ADHD_t1_	0.62	0.47	0.60	0.46	0.74	(0.02)	3.01 ^+^	(0.07)	0.75	(0.02)	2.89	(0.06)
ADHD_t2_	0.38	0.39	0.41	0.40								
Antisocial_t1_	0.17	0.14	0.19	0.18	0.56	(0.01)	0.06	(0.00)	0.59	(0.02)	0.10	(0.00)
Antisocial_t2_	0.11	0.10	0.11	0.09								
**Quality of life**												
Mother_t1_	86.35	7.11	80.07	9.20	2.94 ^+^	(0.07)	2.50	(0.06)	3.82 ^+^	(0.10)	3.90 ^+^	(0.09)
Mother_t2_	64.60	9.86	64.17	8.02								
Child_t1_	74.15	12.96	74.44	9.45	4.52 *	(0.11)	0.83	(0.02)	4.60 *	(0.12)	0.77	(0.02)
Child_t2_	63.25	9.77	62.26	9.05								

Note. ^+^ *p* < 0.10, * *p* < 0.05. VSD = ventricular septal defect. Neurodevelopment: t1, Intelligence and Development Scales, IDS [67]; t2, Wechsler Intelligence Scale for Children—Fifth Edition, WISC-V [63]; Cognitive = cognitive development: *M* = 100, *SD* = 15; language: z-standardization was used in order to transfer the IDS and WISC-V values into a common unit, *M* = 0, *SD* = 1. Maternal psychopathology ratings at t1 and t2: Diagnostic System for Psychiatric Disorders according to ICD-10/DSM-IV, DYSIPS [18]—0, ‘not at all’; 1, ‘a little bit’; 2, ‘to a great extent’; 3, ‘particularly’. ADHD = attention deficit/hyperactivity disorder, Antisocial = antisocial behavior. Quality of life: t1, Revised Quality of Life Questionnaire [68]; t2: German version of the Kidscreen-10 questionnaire [69] (0 to 100% quality of life). Sample size (*n*): VSD: Cognitive/Language *n* = 18; maternal psychopathology ratings, Depression/Anxiety/Antisocial *n* = 20, ADHD *n* = 23; quality of life, Mother *n* = 20, Child *n* = 16. Controls: Cognitive/Language *n* = 23; maternal psychopathology ratings, Depression/ADHD/Antisocial *n* = 23, Anxiety *n* = 22; quality of life, Mother/Child *n* = 23. Covariates—Cognitive/Language: socioeconomic status. Degrees of freedom in rm ANCOVAs (*df_F_*): Cognitive/Language *df_F_* = 36, Depression *df_F_* = 39, Anxiety *df_F_* = 38, ADHD *df_F_* = 42, Antisocial *df_F_* = 39, Quality of life Mother *df_F_* = 39, Quality of life Child *df_F_* = 35. *dfH* = 1. *η*^2^*_p_*, partial eta-squared, effect size measure: ≥0.01 small effect, ≥0.06 medium effect, ≥0.14 large effect.

**Table 5:** ANCOVA-Tested Differences between the VSD group and Controls. Covariate Maternal Anxiety (Model A_t1/t2_) or Maternal Parenting (Model B_t1/t2_).

	VSD		Controls		Statistics F (*η*^2^*_p_*)			
	*M*	*SD*	*M*	*SD*				
**Adolescent‘s psychopathology rating**					group		group × anxiety t1	
Depression_t2_	0.31	0.33	0.34	0.36	1.36	(0.03)	1.31	(0.03)
Anxiety_t2_	0.34	0.42	0.38	0.35	0.33	(0.01)	0.27	(0.01)
ADHD_t2_	0.40	0.36	0.16	0.22	0.00	(0.00)	0.32	(0.01)
Antisocial_t2_	0.19	0.20	0.18	0.10	0.92	(0.02)	1.03	(0.03)
							group × anxiety t2	
Depression_t2_	0.32	0.32	0.34	0.36	0.09	(0.00)	0.10	(0.00)
Anxiety_t2_	0.34	0.41	0.38	0.35	0.03	(0.00)	0.02	(0.00)
ADHD_t2_	0.40	0.36	0.16	0.22	0.93	(0.02)	0.39	(0.01)
Antisocial_t2_	0.19	0.20	0.18	0.10	3.26 ^+^	(0.07)	3.34 ^+^	(0.08)
							group × parenting t1	
Depression_t2_	0.31	0.32	0.33	0.37	6.56 *	(0.15)	6.87 *	(0.15)
Anxiety_t2_	0.34	0.42	0.38	0.36	3.98 ^+^	(0.10)	4.42 *	(0.11)
ADHD_t2_	0.40	0.36	0.16	0.23	1.61	(0.04)	0.76	(0.02)
Antisocial_t2_	0.19	0.20	0.17	0.10	1.64	(0.04)	10.57	(0.04)
							group × parenting t2	
Depression_t2_	0.34	0.32	0.34	0.36	1.23	(0.03)	1.33	(0.03)
Anxiety_t2_	0.36	0.42	0.38	0.35	0.09	(0.00)	0.12	(0.00)
ADHD_t2_	0.44	0.38	0.16	0.22	0.11	(0.00)	0.04	(0.00)
Antisocial_t2_	0.19	0.20	0.18	0.10	0.30	(0.01)	0.28	(0.01)
**Cortisol Adolescent**					group		group × anxietyt1	
	0.18	0.67	0.67	0.60	0.03	0.00	0.03	(0.00)
							group × anxiety t2	
	0.17	0.64	0.67	0.60	0.07	0.00	0.00	(0.00)
							group × parenting t1	
	0.18	0.67	0.65	0.61	2.58	0.08	3.73 ^+^	(0.12)
							group × parenting t2	
	0.27	0.62	0.67	0.60	3.09 ^+^	0.10	3.94 ^+^	(0.12)

Note. ^+^ *p* < 0.10, * *p* < 0.05. Adolescent’s psychopathology rating at t2: Diagnostic System for Psychiatric Disorders according to ICD-10/DSM-IV, DYSIPS [18]—0, ‘not at all’; 1, ‘a little bit’; 2, ‘to a great extent’; 3, ‘particularly’. Sample size (*n*): VSD: psychopathology self-ratings, Depression *n* = 18–20, Anxiety *n* = 19–21, ADHD *n* = 18–22, Antisocial *n* = 19–21, Cortisol *n* = 12–14; Controls: psychopathology self-ratings, Depression *n* = 23–24, Anxiety *n* = 21–22, ADHD *n* = 23–24, Antisocial *n* = 23–24, Cortisol *n* = 12–14. Degrees of freedom in ANCOVAs (*df_F_*): Depression *df_F_* = 38–40, Anxiety *df_F_* = 37–39, ADHD *df_F_* = 38–40, Antisocial *df_F_* = 39–41, Cortisol *df* = 28–30. *dfH* = 1. Adolescent cortisol at t2: ln-transformed hair cortisol concentration. VSD = ventricular septal defect. ADHD = attention deficit/hyperactivity disorder, Antisocial = antisocial behavior. *η*^2^*_p_*, partial eta-squared, effect size measure: ≥0.01 small effect, ≥0.06 medium effect, ≥0.14 large effect.

## Data Availability

Data are available upon reasonable request.
